# Role of signal transduction pathways in IL‐1β‐induced apoptosis: Pathological and therapeutic aspects

**DOI:** 10.1002/iid3.762

**Published:** 2023-01-13

**Authors:** Peixuan Wang, Hong Qian, Manxue Xiao, Jingwen Lv

**Affiliations:** ^1^ Department of Pediatric Dentistry, Stomatological Hospital Southern Medical University Guangzhou China

**Keywords:** apoptosis, interleukin‐1β, MAPKs signaling pathway, NF‐κB signaling pathway, PI3K/Akt signaling pathway

## Abstract

**Background:**

Interleukin‐1β (IL‐1β) is a pro‐inflammatory cytokine mainly produced by monocytes and macrophages with a wide range of biological effects. Evidence has shown that IL‐1β plays a vital role in the process of apoptosis; however, the specific mechanisms, by which IL‐1β induces apoptosis, vary due to different cellular and experimental conditions. Therefore, this present reviewstudy aimed to systematically review the association between the molecular mechanisms of IL‐1β‐induced apoptosis in pathological processes and the role of signaling pathways. This article also sought to briefly investigate the potential of signaling pathway‐targeted therapy in the prevention and treatment of disease.

**Methods:**

This is a literature review article. The present discourse aim is first to scrutinize and assess the available literature on IL‐1β and apoptosis. The relevant studies using the keywords of “IL‐1β‐induced apoptosis” and “signaling pathways” were searched in the databases of PubMed, Scopus, Google Scholar, and Web of Science. Gathered relevant material, and extracted information was then assessed.

**Results:**

IL‐1β can induce apoptosis in various types of cells under different external stimuli via the mitochondrial pathway, death receptor pathway and endoplasmic reticulum pathway, and that the different pathways are often interconnected. The NF‐kB signaling pathway, p38MAPK, and JNK signaling pathways mainly play a proapoptotic part, and the ERK1/2 pathway has a bidirectional role in regulating apoptosis, while activation of the PI3K‐Akt signaling pathway can inhibit apoptosis.

**Conclusion:**

This review indicates that IL‐1β‐induced apoptosis plays an important role in pathogenesis and development of pathology of many inflammatory diseases. Elucidating the role of the signaling pathways will aid the development of targeted therapeutic treatments.

## Graphical abstract

Evidence has shown that interleukin‐1β (IL‐1β) plays a vital role in the process of apoptosis. This paper reviews the molecular mechanisms of IL‐1β‐induced apoptosis in pathological processes, focusing on the role of signaling pathways. This article also briefly describes the potential of signal pathway‐targeted therapy in the prevention and treatment of disease, using the application of signal pathways in the treatment of disc degeneration and osteoarthritis as examples.

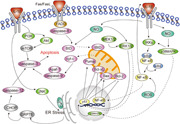

## 1 INTRODUCTION

Interleukin‐1β (IL‐1β) is a quintessential pro‐inflammatory cytokine expressed primarily by monocytes and macrophages. The biological functions of IL‐1β mainly include mediating inflammatory responses and innate immunity,^1^ and IL‐1β's ability to induce DNA methylation is closely related to the occurrence and development of tumors.^2^, ^3^ In addition, with the discovery of the relationship between osteoarthritis and IL‐1β‐induced chondrocyte apoptosis,^4^ there is increasing evidence that IL‐1β can induce apoptosis, such as cardiomyocyte,^5^ nucleus pulposus cells,^6^ human umbilical vein endothelial cells,^7^ β‐cells,^8^ and fibrosus cells.^9^

Apoptosis is the autonomous and orderly death of cells under certain physiological or pathological conditions, which is controlled by a series of activation, expression, and regulation of genes. Currently, the three well‐known pathways for apoptosis are mitochondria pathway, death receptor pathway, and endoplasmic reticulum stress pathway, whose ability to mediate IL‐1β‐induced apoptosis have been proven by studies. The difference between the mechanism of the three pathways in regulating the apoptosis process may result from the type, origin, growth environment of cells, and the external factors to initiate apoptosis, based on which the signal transduction pathways involved also differ.

### 1.1 IL‐1β overview

#### 1.1.1 The interleukin‐1 superfamily

The interleukin‐1 superfamily includes IL‐1α, ‐1Ra, ‐18, ‐33, ‐36Ra, ‐36α, ‐36β, ‐3 6γ, ‐37, and ‐38, of which IL‐1β is one of the earliest discovered and most important members. The IL‐1β gene is encoded by seven exons and six introns. The abundance of AU sequence on the 3′ untranslated zone of the seventh exon causes IL‐1β to be extremely unstable and easily degradable by enzymes,^10^ which is a prerequisite for IL‐1β to be cleavable.

#### 1.1.2 Activation and action of IL‐1β

IL‐1β is not through the classical endoplasmic reticulum‐Golgi secretion pathway but sequential activation of vesicular or gasdermin d‐mediated secretory pathways.^11^

In the cytoplasm, there is usually inactive pro‐interleukin‐1 (pro‐IL‐1β), which is the processed by the IL‐1β‐converting enzyme (ICE) and converted into active and mature IL‐1. The mature IL‐1βthen binds to the type 1 IL‐1 receptor (IL‐1R1) to exert biological functions.

### 1.2 Molecular mechanism of IL‐1β‐induced apoptosis

#### 1.2.1 Mitochondria pathway

Mitochondria are organelles critical to the process of apoptosis. In experiments related to IL‐1β‐induced apoptosis of nucleus pulposus cells^12^ and intervertebral disc cells^13^ changes in protein expression levels of Bcl‐2, Bax, caspase‐9, and caspase‐3 can be detected, so it is inferred that IL‐1β significantly increases the proapoptotic protein Bax and decreases the antiapoptotic protein Bcl‐2.^6^, ^14^ The ratio of Bax to Bcl‐2 affects the mitochondrial membrane potential leaving the mitochondrial permeability transition pore open and the mitochondria highly impaired.^15^ Then cytochrome‐c (cyt‐c) is released into the cytoplasm^16^ triggering the downstream caspase‐9‐mediated endogenous apoptotic pathway.^17^ Hydrogen sulfide^18^ and appropriate mechanical stress^19^ were found to improve apoptosis that is associated with mitochondrial dysfunction.

#### 1.2.2 Death receptor pathway

In recent years, Hui et al.^8^ found that the IL‐1β/Fas/caspase‐8 apoptotic pathway plays a role in amyloid‐induced β‐cell apoptosis in an in vitro cultured human islet amyloid models. Similarly, Park et al. found that the increase in IL‐1β levels is closely related to the upregulation of Fas and the activation of caspase‐8.^20^ Therefore, it can be assumed that IL‐1β can induce the exogenous apoptosis pathway mediated by death receptors. Structural changes occur after the binding of Fas with the ligand FasL. Then, the activated Fas binds with the death domain, which aggregates Fas death domain‐associated protein to form a dimer. The dimer then further activates caspase‐8 triggering a downstream caspase‐3 cascade reaction, which finally leads to apoptosis. In addition, Kim et al. found that caspase‐8 cleaved the proapoptotic protein Bid into tBid, which is accompanied by the release of cyt‐c and the production of reactive oxygen species (ROS). Kim's discovery linked the death receptor pathway and the mitochondrial pathway.^21^

#### 1.2.3 Endoplasmic reticulum pathway

Dong et al.^22^ found that the process of IL‐1β‐induced neuronal apoptosis was accompanied by Ca^2+^ imbalance and upregulation of endoplasmic reticulum stress‐related proteins GRP78 and CHOP. Li et al.^23^ also found that IL‐1β‐induced chondrocytes underwent endoplasmic reticulum stress‐mediated apoptosis, along with elevated expression of GRP78, CHOP, and cleaved caspase‐12. All of the above experiments demonstrate that IL‐1β can induce endoplasmic reticulum stress (ERS)‐mediated apoptosis through the GRP78/CHOP/caspase‐12 pathway, the process of which often requires the involvement of Ca^2+.^ ^24^ Recently, it has been found that drugs targeting this pathway are effective in reducing endoplasmic reticulum stress and IL‐1β‐induced apoptosis, thereby alleviating inflammatory disease.^25^

For IL‐1β to induce apoptosis, a single pathway can work in a stand‐alone fashion or interact with other pathways to take effect collaboratively. Some scholars suggest that IL‐1β upregulates Bax, cyt‐c, caspase‐9, and caspase‐3 to induce apoptosis via the mitochondrial damage pathway.^9^, ^15^ Conversely, Park et al.^26^ stated that apoptosis of intervertebral disc cells occurs only through the membrane receptor pathway and that the mitochondrial injury pathway is not involved in this process. However, Heyde's study found that both the membrane receptor pathway and the mitochondrial injury pathway are involved in apoptosis of intervertebral disc cells.^27^ Additionally, IL‐1β not only activates the caspase‐dependent pathway but also indirectly promotes the occurrence of apoptosis by inhibiting the expression of members of the inhibitors of apoptosis proteins family.^28^ The different findings may be due to different concentrations and duration of action of IL‐1β, and the specific mechanisms are to be validated in more different types of cells.

### 1.3 Signaling pathways involved in the regulation of IL‐1β‐induced apoptosis

**Figure 1:**
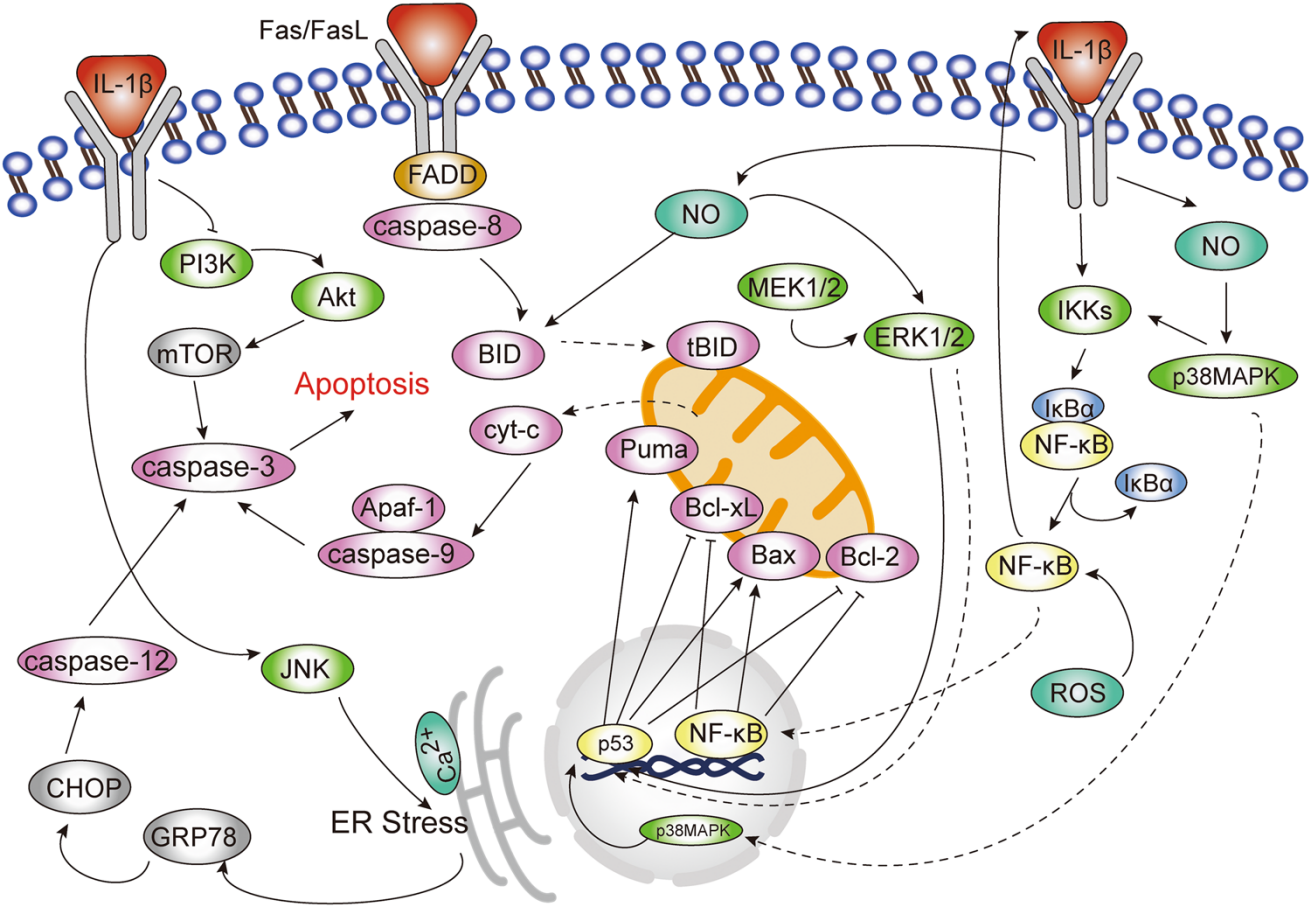
Role of signal transduction pathways in interleukin‐1β (IL‐1β)‐induced apoptosis. The nuclear factor‐κB (NF‐κB) signaling pathway, p38 mitogen‐activated protein kinase (p38MAPK), and c‐Jun N‐terminal kinase (JNK) signaling pathways mainly play a proapoptotic part, and the extracellular signal‐regulated protein kinase (ERK1/2) pathway has a bidirectional role in regulating apoptosis, while activation of the PI3K‐Akt signaling pathway can inhibit apoptosis.

IL‐1β‐induced apoptosis is the result of the interaction and joint regulation of multiple signaling pathways including nuclear factor‐κB (NF‐κB) signaling pathway, mitogen‐activated protein kinases (MAPKs) signaling pathway, and phosphoinositol‐3 kinase (PI3K)/Akt signaling pathway, etc. Among them, NF‐κB signaling pathway, p38 mitogen‐activated protein kinase (p38MAPK) signaling pathway, and c‐ Jun N‐ terminal kinase (JNK) signaling pathway mainly play a pro‐apoptotic role, and the extracellular signal‐regulated protein kinase (ERK) 1/2 pathway has a bidirectional role in regulating apoptosis, while activation of the PI3K‐Akt signaling pathway can inhibit apoptosis (Figure 1).

#### 1.3.1 NF‐κB signaling pathway

NF‐κB has been reported to be involved in regulation of the IL‐1β‐induced apoptosis in different diseases.^29^, ^30^, ^31^, ^32^ There are two different pathways for NF‐κB signaling activation: the classical pathway and the alternative pathway.^33^ IL‐1β acts as an immune mediator to induce the classical NF‐κB pathway, where IL‐1β and IL‐1R binds to activate the IκB kinases (IKKs) complex. IKKs then degrade downstream inhibitor of NF‐κB (IκB) molecules to translocate NF‐κΒ into the nucleus to affect the transcription of related target genes.^34^, ^35^ Yun et al. used the inhibitor PS1145 to verify that activation of NF‐κB is essential to the process of IL‐1β‐inducing apoptosis,^32^ and if phosphorylation of NF‐κB p65 is inhibited or reversed,^36^, ^37^, ^38^ the activation of NF‐κB and nuclear transposition are affected,^39^, ^40^, ^41^ thus, exerting antiapoptotic effects. To further explore the specific mechanism, Guo et al. found that the expression of NF‐κB‐regulated gene products and the level of proapoptotic protein caspase‐3 were significantly increased in the IL‐1β treatment group of synovial cells in rheumatoid arthritis rats, while the expression of antiapoptotic proteins Bcl‐2 and Bcl‐xL was significantly decreased.^31^

NF‐κB is central to the action of many pro‐inflammatory factors. NF‐κB is activated by pro‐inflammatory factors, which in turn directly or indirectly upregulates their levels to create a positive feedback.^33^ IL‐1β is one of these pro‐inflammatory factors to activate NF‐κB. The activation of NF‐κB upregulates the release of IL‐1β, IL‐6,^42^ and tumor necrosis factor‐α (TNF‐α),^43^ which fuels apoptosis^44^, ^45^ and inflammation.^46^, ^47^ ROS activated by IL‐1β is another pro‐inflammatory factors.^48^ NF‐κB, as a downstream molecule of ROS^49^ when activated, stimulates the release of pro‐inflammatory factors such as cyclo‐oxygenase‐2 (COX‐2), prostaglandin E2 (PGE2), and NO.^50^ Then, the NO in turn activates NF‐κB in a p38MAPK‐dependent manner, forming a positive feedback pathway that promotes apoptosis. Some research focus on reducing the production of ROS and NO, and so on, so as to inhibit IL‐1β‐induced apoptosis and inflammation progression.^51^, ^52^, ^53^, ^54^ However, it has been suggested that the effect of ROS on NF‐κB may be related to the duration of oxidative stress, so the sustained oxidative stress may instead inhibit NF‐κB activity.^55^

#### 1.3.2 MAPKs signaling pathway

Extensive studies have shown that the MAPKs signaling pathway is activated by various physical, chemical, biological, and other stimuli, and plays a key role in apoptosis. Typical MAPK family signaling pathways mainly include ERK, JNK, and p38MAPK. There is extensive traffic between the pathways, and these kinases integrate signals at different sites through transcription‐dependent and transcription‐independent mechanisms, ultimately converging on caspase activation to lead to apoptosis. Tu et al. found that the phosphorylation levels of JNK, p38MAPK, and ERK were significantly higher than those in the control group after IL‐1β stimulation for 48 h.^39^ One of the mechanisms by which drugs can suppress the progression of arthritis is by reducing IL‐1β‐induced phosphorylation of MAPKs.^18^, ^56^ Therefore, it can be assumed that IL‐1β activates the MAPKs pathway, which induces apoptosis.

##### p38MAPK signaling pathway

The p38MAPK signaling pathway is involved in regulating the IL‐1β‐induced apoptosis^57^ with nitric oxide (NO)^58^ and NF‐κB^59^ being important molecules involved in this pathway. Inducible nitric oxide synthase (iNOS) and NO are present in apoptotic cells induced by IL‐1β. iNOS is an upstream signaling molecule for NO. NO can be antiapoptotic or pro‐apoptotic, depending on its concentration, source, and duration.

NO of High concentrations can promote apoptosis in a variety of cells, and the proapoptotic mechanisms may include the following three: (1) NO promotes apoptosis by regulating Bax/Bcl‐2 proteins and upregulating caspase‐3 through a p38/p53‐dependent pathway,^60^, ^61^ while blocking the p38/p53 pathway to prevent IL‐1β‐induced neuronal apoptosis.^62^ Upregulation of p53 specifically involves two pathways: first, p38 phosphorylates the upstream kinase of IκB, which activates NF‐κB and increases p53 transcription; second, p38 can directly phosphorylate Serine 15 residues of p53, thereby stabilizing p53 protein content.^63^ (2) NO forms ONOO‐ with O2‐, leading to mitochondrial dysfunction, cleavage of Bid, and activation of Fas to enable cascade reaction with downstream caspase‐8.^64^ (3) In an in vitro experiment on rat pheochromocytoma cells, Li et al. found that NO upregulated COX‐2 expression by enhancing the binding of activator protein‐1 (AP‐1) to DNA leading to p53 accumulation and apoptosis.^65^

The antiapoptotic mechanisms of NO of low concentrations include inducing the formation of heat shock protein 70,^66^ enhancing Bcl‐2 expression,^67^ and directly inhibiting caspase‐8 protease activity,^68^ and so on. In mammals, the antiapoptotic properties of NO are often associated with cyclic guanylate cyclase (cGMP) dependence.^69^ Although NO is associated with apoptosis, the available studies on the involvement of NO in the regulation of IL‐1β‐induced apoptosis are mainly related to p53. So, it remains unclear whether the other related mechanisms mentioned above play a role in IL‐1β‐induced apoptosis.

##### JNK signaling pathway

IL‐1β induces JNK signaling,^37^ but intervention with the drug^70^, ^71^ or the JNK inhibitor SP600125^72^, ^73^ can inhibit IL‐1β‐induced JNK1/2 activation and apoptosis. A range of evidence links the JNK signaling pathway to IL‐1β‐induced apoptosis. The specific mechanisms may be: (1) The JNK pathway may be an upstream pathway of endoplasmic reticulum stress. Previous studies have shown that IL‐1β induces endoplasmic reticulum stress and apoptosis in pancreatic β‐cells in a JNK‐dependent manner.^74^ (2) Promote apoptosis via the JNK/p53 pathway.^75^ Ling et al. verified that lower JNK levels and reduced p53 phosphorylation were associated with inhibition of neuronal apoptosis.^76^ One explanation might be that the phosphorylated JNK activates p53, which phosphorylates itself at the proline‐rich structural domain Threonine 81 site. Then Phosphorylated p53 upregulates the expression of various proapoptotic target genes such as Puma^77^ and Bax,^78^ while decreasing the expression of antiapoptotic genes such as Bcl‐2^72^ and Bcl‐xL, resulting in cyt‐c release and apoptosis. However, in some cases, p53 can promote apoptosis independently of JNK activation.^79^ At present, it is unclear whether JNK nondependent activation of p53 is associated with the process of IL‐1β‐induced apoptosis.

##### ERK signaling pathway

The ERK1/2 pathway has both antiapoptotic and proapoptotic effects, depending on the experimental conditions and/or cell types. One of the mechanisms involved in IL‐1β‐induced apoptosis may be through the MEK/ERK signaling pathway,^80^ which increases the expression of Bax and caspase‐3 and decreases the expression of Bcl‐2. Inhibition of ERK1/2 may block IL‐1β‐induced mitochondrial damage and apoptosis.^81^ Upregulation of p53 levels may also be one of the mechanisms. The experiments of Sun et al.^82^ and Lee et al.^83^ found that ERK1/2, an upstream signal of p53, induces apoptosis by activating the ERK1/2‐p53 pathway and upregulating caspase‐3. However, it has also been shown that after activation of ERK1/2 by NO, ERK1/2 instead inhibits p53 phosphorylation thereby protecting chondrocytes from apoptosis.^84^ All of the above experiments can demonstrate that the ERK1/2‐p53 pathway plays an important role in apoptosis, but it is still unclear how this pathway affects IL‐1β‐induced apoptosis so further experimental studies are needed.

##### PI3K/Akt signaling pathway

In recent years, there has been increasing evidence that regulation of the cell cycle through the PI3K/Akt signaling pathway can inhibit IL‐1β‐induced apoptosis and inflammatory.^85^, ^86^ First, Cai et al. found that compared to normal cells and tissues, apoptosis was increased in IL‐1β‐treated articular chondrocytes accompanied by downregulation of PI3K mRNA levels,^4^ which demonstrated that PI3K is associated with apoptosis. Next, Guo et al. found that 17β‐estradiol attenuated IL‐1β‐induced early apoptosis in nucleus pulposus cells by increasing the expression of activated mechanistic target of rapamycin (mTOR) and decreasing the expression of activated caspase‐3, in intervertebral disc lesions.^87^ Yan et al.^88^ showed that IL‐1β‐induced chondrocyte apoptosis is due to the weakening of the PI3K/Akt/mTOR pathway and that activation of this pathway is protective against apoptosis. In addition, Xu et al.^89^ suggested that the suppression of apoptosis by drugs through the PI3K/Akt pathway may also be associated with altered mitochondrial membrane potential.

### 1.4 Targeted therapeutic applications

IL‐1β has been shown to play an integral role in the pathology of many inflammatory diseases, including the induction of apoptosis and the mediation of inflammatory responses. The clarification of the mechanisms of disease progression provides the molecular basis for research into new drugs and opens up new rationales. Signaling pathway‐targeted therapies have been studied more centrally in the treatment of disc degeneration and osteoarthritis, and as such are illustrated below as an example.

A recent review revealed that the activation of the PI3K/Akt signaling pathway alleviated intervertebral disc degeneration and focused attention on the therapeutic effects of estrogen.^90^ Resveratrol increased the ratio of IL‐1β‐induced p‐Akt protein expression to Akt protein expression in myeloid cells with partial inhibition of apoptosis‐related molecules, and its protective effect against IL‐1β‐induced apoptosis in NP cells was diminished when the inhibitor LY294002 was further added.^14^ Likewise, other studies also suggested the effect of 17β‐estradiol,^91^ proanthocyanidins,^92^ and omentin‐1^93^ against apoptosis induced by IL‐1β in NP cells via the PI3K/Akt pathway. In the other hand, the NF‐κB pathway is also an effective intervention site. IL‐1β‐mediated phosphorylation of p65 and translocation of NF‐κB were attenuated by the addition of berberine, indicating that activation of the NF‐κB pathway was inhibited by berberine. In addition, berberine prevented the degradation of the extracellular matrix of nucleus pulposus cells and has therapeutic potential in the prevention or treatment of intervertebral disc degeneration.^40^

In IL‐1β‐induced chondrocyte injury, NaHS partially inhibited IL‐1β‐induced phosphorylation of the MAPK cascade, significantly reversing apoptosis associated with mitochondrial dysfunction^18^; LMP‐1 suppressed the expression of p‐p65 and p‐JNK, reducing apoptosis by regulating NF‐κB and MAPK/JNK pathways.^71^ Tricetin repressed the expression of MMPs and IL‐1β‐induced NO and PGE2 production, while markedly attenuating IL‐1β‐induced phosphorylation of JNK and p38, exerting antiapoptotic effects via Bax/Bcl‐2/caspase‐3.^94^ Tetramethylpyrazine protects chondrocytes by reducing endoplasmic reticulum stress through inhibition of IL‐1β‐induced GRP78 and CHOP expression,^95^ which is likely to be a potential therapeutic agent for osteoarthritis.

Apart from the currently reported drugs, numerous other biotherapeutic studies have been published. circular RNAs^96^, ^97^, ^98^ and Long noncoding RNAs^99^, ^100^, ^101^ are currently a hot topic of research, and their antiapoptotic mechanisms are not identical to those of traditional signal pathways, pending further in‐depth studies at the molecular level.

## 2 CONCLUSION AND PROSPECTS

This paper reviews various literature and studies to indicate that IL‐1β can induce apoptosis in various types of cells under different external stimuli via the mitochondrial pathway, death receptor pathway and endoplasmic reticulum pathway, and that the different pathways are often interconnected. The NF‐κB signaling pathway, p38MAPK, and JNK signaling pathways mainly play a proapoptotic part, and the ERK1/2 pathway has a bidirectional role in regulating apoptosis, while activation of the PI3K‐Akt signaling pathway can inhibit apoptosis. As apoptosis is a complex process, the molecules, substrates, mechanisms of action and regulation of the signal transduction pathway remain to be further investigated. The study of signal pathways has led to a clearer understanding of the pathophysiology of disease and has provided a theoretical basis and new ideas for the development of targeted therapeutics that interfere specifically with the expression of molecules in one or more signal pathways to stop disease progression accurately and effectively. We look forward to a future where the treatment of IL‐1β and its signal pathways may become a new target for the treatment of inflammatory diseases, and inhibitors of the caspase downstream pathway are promising new loci for treatment. At present, many of these targeted therapeutics remain in in vitro models with a long way to go before they can be used clinically. In addition, the research of normal physiological activity is no less important than the study of the onset of disease.

In the future, it may be possible to explore the role of IL‐1β in inducing apoptosis during physiological processes, such as physiological root resorption in deciduous teeth, based on an understanding of the IL‐1β signal pathway, which has great potential for preventive applications.

## CONFLICT OF INTEREST

The authors declare no competing interests.

## Data Availability

All the information included in this manuscript is available upon request by contact with the corresponding author.
